# Electrochemical Properties of an Na_4_Mn_9_O_18_-Reduced Graphene Oxide Composite Synthesized via Spray Drying for an Aqueous Sodium-Ion Battery

**DOI:** 10.3390/nano7090253

**Published:** 2017-09-02

**Authors:** Fuxing Yin, Zhengjun Liu, Yan Zhao, Yuting Feng, Yongguang Zhang

**Affiliations:** 1 School of Materials Science & Engineering, Research Institute for Energy Equipment Materials, Tianjin Key Laboratory of Materials Laminating Fabrication and Interface Control Technology, Hebei University of Technology, Tianjin 300130, China; yinfuxing@hebut.edu.cn (F.Y.); lzj125@126.com (Z.L.); 2 Synergy Innovation Institute of GDUT, Heyuan 517000, China; 18222385469@163.com

**Keywords:** aqueous sodium-ion battery, cathode, Na_4_Mn_9_O_18_, energy storage materials

## Abstract

An aqueous sodium ion battery (ASIB) with metal Zn as anode and Na_4_Mn_9_O_18_-reduced graphene oxide (Na_4_Mn_9_O_18_-RGO) as cathode has been developed. In this work, spherical Na_4_Mn_9_O_18_-RGO composite particles were prepared via spray drying. The aqueous battery exhibits stable cyclability and high specific capacities. Typically, a high initial discharge capacity of 61.7 mAh·g^−1^ is attained at a high current rate of 4 C, and a stabilizing reversible capacity of 58.9 mAh·g^−1^ was obtained after 150 cycles. The network interlaced by RGO sheets provided fast electron conduction paths and structural stability to accommodate the mechanical stresses induced by sodium insertion and extraction, so the Na_4_Mn_9_O_18_-RGO electrode displayed superior electrochemical performance in the ASIB.

## 1. Introduction

Recently, sodium-ion batteries (SIBs) have been regarded as promising candidates to lithium ion batteries (LIBs) due to the low cost, relative abundance of sodium [1,2], and analogous intercalation chemistry between sodium and lithium. However, some intrinsic drawbacks still exist in SIBs such as the slower diffusion efficiency in the Na-host materials due to the larger atomic mass of sodium, huge volume expansion, and serious pulverization of electrodes [3,4,5].

Thence, in order to meet the demand of good electrochemical performance, lots of SIB electrode materials have been developed. Various Na-Mn oxides such as NaMn_1/3_Ni_1/3_Co_1/3_PO_4_ [6], Na*_x_*Mn_5_O_10_ [7], Na_2_Mn_5_O_10_ [8], and Na_4_Mn_9_O_18_ have been extensively researched as active materials in SIB systems due to the presence of the extensive channels for Na ion intercalation and deintercalation [9]. Among them, Na_4_Mn_9_O_18_ exhibits the MnO_6_ octahedra and MnO_5_ square cone, which are able to constitute the wide S-type double tunnels for Na ion diffusion [10]. Whitacre et al. reported the Na_4_Mn_9_O_18_ as a positive electrode material for an aqueous sodium ion battery (ASIB), and this material cathode displayed a capacity of 45 mAh·g^−1^ at a current density of C/8 rate [11]. However, Na_4_Mn_9_O_18_ displayed poor storage capability and cyclability owing to the large volume expansion during the intercalation/deintercalation of large Na^+^, resulting in a gradual deterioration of dynamics and structures [12]. Encapsulating Na_4_Mn_9_O_18_ into an elastic carbon matrix offers an ideal strategy to provide an electron pathway for individual particles while limiting surface reactions and reducing the stress associated with the volume change upon Na^+^-ion insertion/extraction [13]. In 2012, hybrid aqueous lithium ion batteries with excellent cyclability and rate capability based on a Zn metal anode and an LiMn_2_O_4_ cathode was reported by P. Chen’s group [14]. Zn metal was chosen as an anode because of its abundance, low cost, and low equilibrium potential [15]. While referring to the literature corresponding to the similar system using a metallic Zn anode, the reversibility of the battery is reported to be a bit of a challenge in the presence of additives [16,17]. The environment of electrolyte has a great effect on the cyclic performance of zinc. Moreover, the acidic electrolyte is obviously different from the alkaline or neutral electrolytes [14].

The spray drying method possesses multitudinous strong points for controlling particle size and morphology, leading to a shortening of the evaporation time of the precursor and homogeneous composition [18]. Spray drying has been applied in the synthesis of numerous LIB cathodes, but it is rarely studied in SIB cathodes [19]. Herein, for the first time, we synthesized a novel Na_4_Mn_9_O_18_-reduced graphene oxide (Na_4_Mn_9_O_18_-RGO) composite with regular morphology and homogeneous composition via spray drying. Furthermore, we have investigated its potential as a positive electrode in an ASIB combining a Zn metal anode and an optimized Na^+^/Zn^2+^ mixed-ion electrolytes.

## 2. Materials and Methods 

### 2.1. The Preparation of Na_4_Mn_9_O_18_-RGO Precursors by the HSCR Method

The Na_4_Mn_9_O_18_-RGO composite precursors were prepared by a hydrothermal soft chemical reaction (HSCR) method with high NaOH concentration [20]. Firstly, 16.65 mL of a 2 mg/mL aqueous colloidal suspension of graphene oxide (GO) prepared by the Hummer’s method [21] was added to 25 mL of a 0.28 M MnSO_4_ aqueous solution while stirring. An equal volume of aqueous solution containing 3.0 M NaOH and 0.1 M KMnO_4_ was then added into the above solution, and dark brown precipitates were quickly produced. The precipitate was washed once by deionized water and aged for 24 h to obtain Na-birnessite-GO precursors. Secondly, 4.0 g wet Na-birnessite-GO samples were added to 100 mL of a 15 M NaOH solution during a 30 min magnetic stirring period, and a dark brown suspension was then formed. Finally, the suspension was heated at 180 °C for 18 h using a 150 mL stainless steel autoclave with a Teflon liner. The resulting product was washed repeatedly with de-ionized water and dried at 60 °C in the air. 

### 2.2. The Preparation of Na_4_Mn_9_O_18_-RGO by Spray Drying

The spray drying system consists of atomizer, quartz reactor, and particle collector [22]. The prepared slurry was spray-dried by a spray drier system (HOLVES). The solution was transferred to a pneumatic atomizing nozzle by a peristaltic pump at 6 mL/min to form droplets. The moisture of the droplets was quickly evaporated after atomization in hot air. The entrance and exit temperatures of a spray drying machine were 200 and 110 °C, respectively. The feed solution was prepared with 0.6 g of prepared Na_4_Mn_9_O_18_-RGO precursors in 200 mL of deionized water and mixed under mild ultra-sonication for 20 min to form a brown suspension. Then, the above suspension was spray-dried with an atomizing pressure of 0.8 MPa to produce the spray-dried powder.

**Figure 1:**
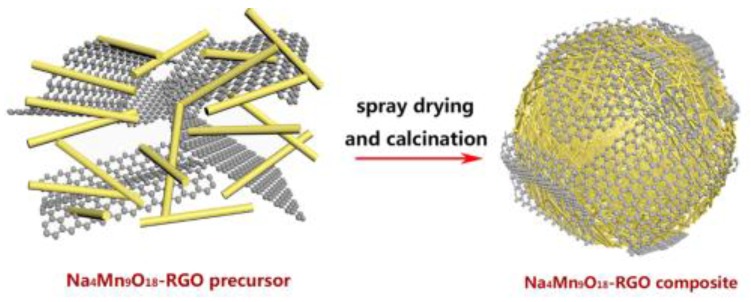
The schematic diagram of Na_4_Mn_9_O_18_-RGO composite by spray drying.

Finally, the sample was further calcined under an Ar atmosphere for 2 h at 350 °C to form Na_4_Mn_9_O_18_-RGO composite particles, as shown in the schematic diagram in Figure 1. Reference Na_4_Mn_9_O_18_ particles without RGO was prepared following the same conditions.

### 2.3. Materials Characterization

The crystalline structure of as-prepared samples was characterized by X-ray diffraction (XRD, D8 Discover, Bruker, Germany) employing Cu Kα radiation. Raman spectra were attained with an Ar-ion laser of 532 nm using the inVia Reflex Raman imaging microscope system. Thermogravimetric analysis (TG, SDT Q-600, TA Instruments-Waters LLC, DE19720, Newcastle, PA, USA) was carried out from 25 to 1000 °C with a heating rate of 10 °C·min^−1^ under air. Scanning electron microscopy (SEM) analysis conducted on a Hitachi Limited S-4800 scanning electron microscope (Tokyo, Japan). The interior structure and selected area electron diffraction (SAED) of samples were studied using a JEOL JEM-2800 high resolution transmission electron microscope (HRTEM, Tokyo, Japan) at 160 kV. 

### 2.4. Electrochemical Measurements

For preparing cathodes, the Na_4_Mn_9_O_18_-RGO particles were blended with carbon black and polyvinylidene fluoride (PVDF) in a weight ratio of 8:1:1 in *N*-methyl-2-pyrrolidone (NMP). The above slurry was spread onto a graphite foil current collector and dried at 70 °C for 10 h. Disks with a diameter of 15 mm were cut with the active material of 3–3.5 mg. Zinc disks with a diameter of 15 mm were used as anodes. The electrolyte was a solution of 1 M Na_2_SO_4_ and 0.5 M ZnSO_4_ with a pH level of 4 [23]. An absorbed glass mat (NSG Corporation, Tokyo, Japan) was used as a separator [24]. CR2025 cells were assembled in an air atmosphere before electrochemical tests were conducted. A battery tester (Neware, Shenzhen, China) was applied to investigate the charge/discharge cycling performances at different current densities in the voltage range of 1–1.85 V vs. Zn/Zn^2+^. The electrochemical workstation (Princeton, VersaSTAT 4, 50/60 Hz, Ametek, PA, USA) was used to test the cyclic voltammogram (CV) in the voltage range of 1–2 V and the electrochemical impedance spectroscopy (EIS) in the frequency range of 0.01–100 kHz with an amplitude of 10 mV. 

## 3. Result and Discussion

**Figure 2:**
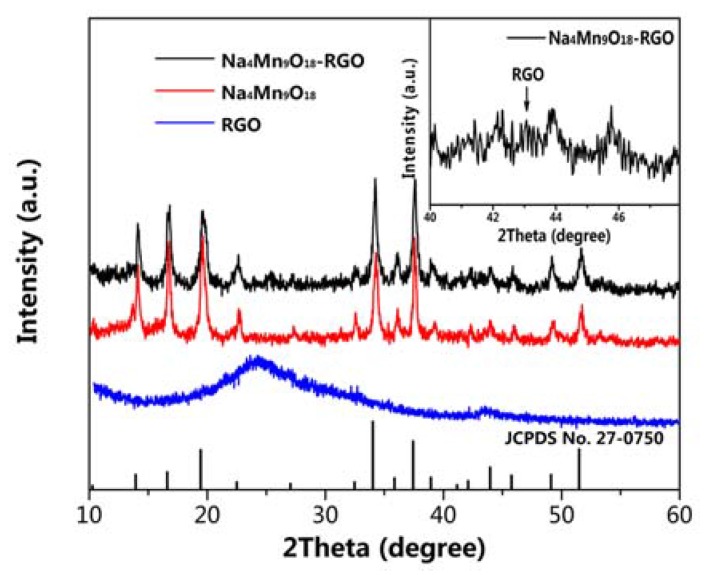
XRD patterns of Na_4_Mn_9_O_18_, Na_4_Mn_9_O_18_-RGO, and RGO.

Figure 2 shows the XRD patterns of Na_4_Mn_9_O_18_ and Na_4_Mn_9_O_18_-RGO particles. The XRD pattern of the Na_4_Mn_9_O_18_ sample are in good agreement with those of orthorhombic Na_4_Mn_9_O_18_ in accordance with JCPDS #27-0750 [25]. The obtained lattice parameters are *a* = 9.1 Å, *b* = 26.34 Å, and *c* = 2.821 Å, which are consistent with previous reports [25,26]. For the Na_4_Mn_9_O_18_-RGO sample, two broad diffraction peaks at around 25.8° and 43.1° can be seen, which are associated with the planes (200) and (100) of the RGO structure [27]. Apart from Na_4_Mn_9_O_18_ and RGO, no foreign peaks from impurities were observed.

**Figure 3:**
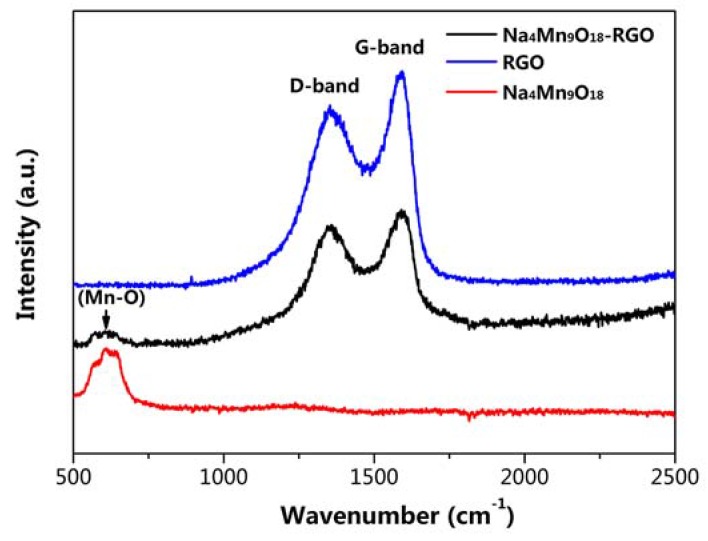
Raman spectrum of Na_4_Mn_9_O_18_, Na_4_Mn_9_O_18_-RGO, and RGO.

Na_4_Mn_9_O_18_ and Na_4_Mn_9_O_18_-RGO particles were further determined by Raman spectra (Figure 3). The broad bands at around 615 cm^−1^ may be associated with stretching vibrations of Mn–O [13]. Compared to the spectrum of Na_4_Mn_9_O_18_, Na_4_Mn_9_O_18_-RGO contained two carbon-related bands at about 1352 (D-band) and 1594 cm^−1^ (G-band). These bands could be due to defective or disorder carbon and graphitic carbon in RGO, respectively [28,29].

**Figure 4:**
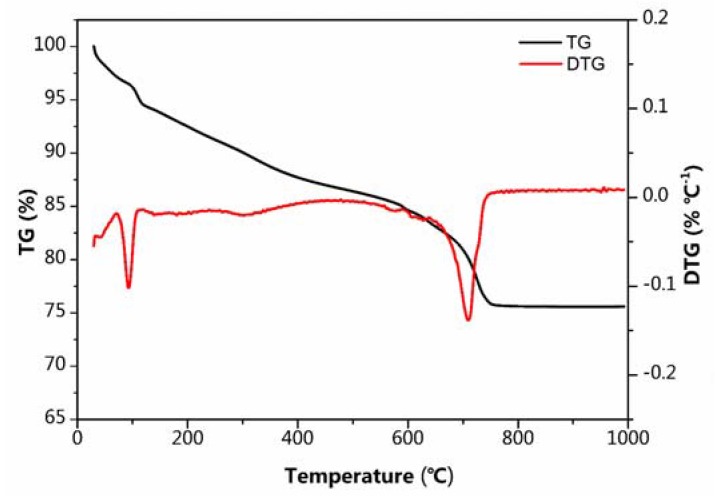
TG-DTG curves of Na_4_Mn_9_O_18_-RGO under an air atmosphere.

The TG and DTG data is collected and shown in Figure 4. The first mass loss about 7% from room temperature to 200 °C is related to the release of physically absorbed water. The second mass loss that appears from 200 to 750 °C is due to the oxidation of C to CO_2_. As we can see, the TG curves of the Na_4_Mn_9_O_18_-RGO, the weight fractions of Na_4_Mn_9_O_18_ and RGO were recorded to be 81.7% and 18.3%, respectively.

**Figure 5:**
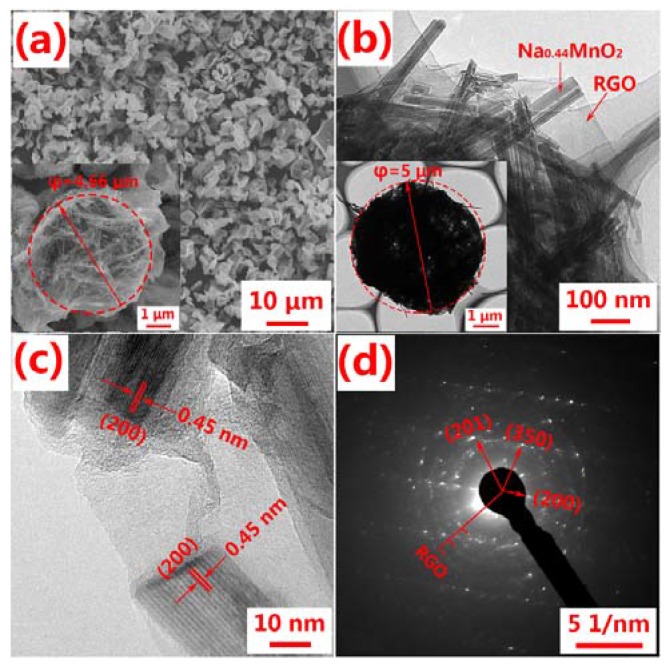
The morphology and structure of the Na_4_Mn_9_O_18_-RGO. (**a**) SEM image; (**b**) TEM image; (**c**) HRTEM image; (**d**) SAED pattern.

Figure 5 shows the morphology and structure of Na_4_Mn_9_O_18_-RGO, which was confirmed by SEM and TEM. As shown in Figure 5a, the Na_4_Mn_9_O_18_-RGO samples present a spherical or ellipsoidal shape with diameters of about 4–6 μm. The inset image of Figure 5a indicates that the Na_4_Mn_9_O_18_-RGO particle has a diameter of 4.66 μm, with rod-like Na_4_Mn_9_O_18_ intimately wrapped in lamellarly structured RGO forming a spherical morphology. In addition, Figure 5b shows clearly the crosslinking state of Na_4_Mn_9_O_18_ and RGO. The rod-shaped Na_4_Mn_9_O_18_ with a diameter of around 50–80 nm are intertwined together by RGO, which will enhance the electrical conductivity of the micron-sized composite. The HRTEM image in Figure 5c shows the vertical lattice fringe is 0.45 nm, which corresponds to the (200) crystallographic plane of Na_4_Mn_9_O_18_ [12]. The SAED pattern in Figure 5d indicates the single crystal nature of Na_4_Mn_9_O_18_ and the polycrystalline of RGO, which is due to the multi-layer stacking.

**Figure 6:**
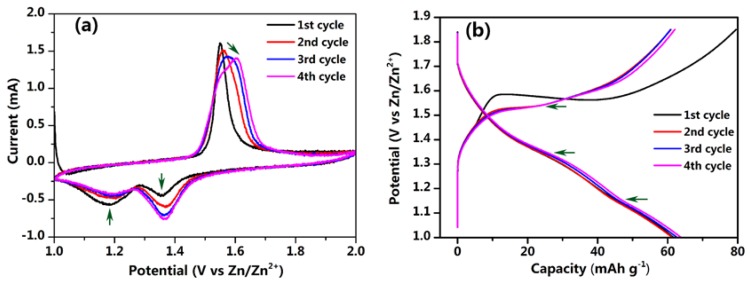
Electrochemical performance of the Na_4_Mn_9_O_18_-RGO electrode (vs. Zn/Zn^2+^). (**a**) CV curves at a rate of 0.1 mV·s^−1^. (**b**) Charge and discharge curves at 4 C.

Figure 6a shows the CV curves of Na_4_Mn_9_O_18_-RGO at a rate of 0.1 mV·s^−1^. There are two reduction peaks at around 1.18–1.19 V and 1.35–1.37 V as well as one oxidation peak at about 1.55–1.6 V during the first four cycles. With increasing cycles, the potentials of the redox peak increase and tend to be stable. In addition, the reduction peak at about 1.18–1.19 V gradually disappeared while the peak at 1.35–1.37 V gradually increased due to the initial activation process of the electrode. The charge and discharge curves are displayed in Figure 6b. The Na_4_Mn_9_O_18_-RGO electrode shows two potential plateaus at about 1.18 and 1.36 V in its discharge process and one plateau at around 1.55 V in its charging cycle, corresponding well with the CV data. The initial coulombic efficiency was only 77.6% due to the high initial charge capacity of 79.5 mAh·g^−1^, which originates from the irreversible side reactions that occurred in the first charge process. The first four discharge curves exhibited an excellent discharge capacity of around 63 mAh·g^−1^ at a high current density of 4 C.

**Figure 7:**
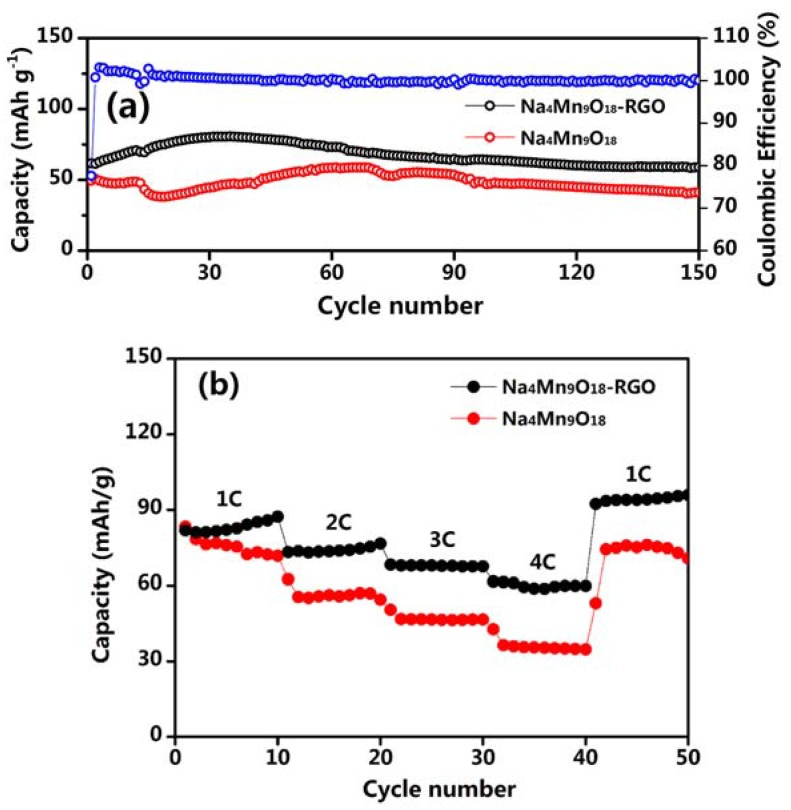
(**a**) Cycling performance of the Na_4_Mn_9_O_18_-RGO and Na_4_Mn_9_O_18_ electrodes at 4 C and coulombic efficiency of the Na_4_Mn_9_O_18_-RGO electrode. (**b**) Rate capability of the Na_4_Mn_9_O_18_-RGO and Na_4_Mn_9_O_18_ electrodes.

Figure 7a presents the cycling performance of the Na_4_Mn_9_O_18_-RGO and Na_4_Mn_9_O_18_ electrodes at 4 C. Both type of electrodes experienced a drop in capacity from the first cycle, which corresponding to the electrodes activation process. The initial discharge capacity of the Na_4_Mn_9_O_18_-RGO electrode was 61.7 mAh·g^−1^, and the maximum capacity was 80.5 mAh·g^−1^ in the 35th cycle. The Na_4_Mn_9_O_18_-RGO and Na_4_Mn_9_O_18_ electrodes both exhibited good cycle stability, maintaining a specific capacity of 58.9 and 41.2 mAh g^−1^ after 150 cycles. Compared with the initial cycle, the two electrodes maintained a capacity of 95.5% and 83.2% after 150 cycles, respectively. For the Na_4_Mn_9_O_18_-RGO electrode, the addition of RGO is necessary for mitigating the low electronic conductivity of Na_4_Mn_9_O_18_ [30]. 

The rate capability of the Na_4_Mn_9_O_18_-RGO and Na_4_Mn_9_O_18_ electrodes are investigated and compared in Figure 7b. As expected, compared with the Na_4_Mn_9_O_18_ electrode, the Na_4_Mn_9_O_18_-RGO electrode represents a better rate performance. The Na_4_Mn_9_O_18_-RGO electrode submits a reversible discharge capacity of around 85 mAh·g^−1^ at a 1 C low current rate. Furthermore, the Na_4_Mn_9_O_18_-RGO electrode can deliver reversible discharge capacities of 75, 68, and 61 mAh g^−1^ at the rates of 2, 3, and 4 C, respectively. Surprisingly, when the current rate was returned to 1 C, the specific discharge capacity of the composite recovered to 94 mAh·g^−1^. This is equivalent to a 110.6% recovery of the initial capacity, corresponding to the electrodes activation process in Figure 7a, suggesting good electrode structure stability of the Na_4_Mn_9_O_18_-RGO electrode. As a comparison, the reversible discharge capacities of the Na_4_Mn_9_O_18_ at each current rate were lower than that of the Na_4_Mn_9_O_18_-RGO. It can be concluded that the network interlaced by RGO sheets provides fast electron conduction pathways and structural stability to accommodate the mechanical stresses induced by sodium insertion and extraction.

**Figure 8:**
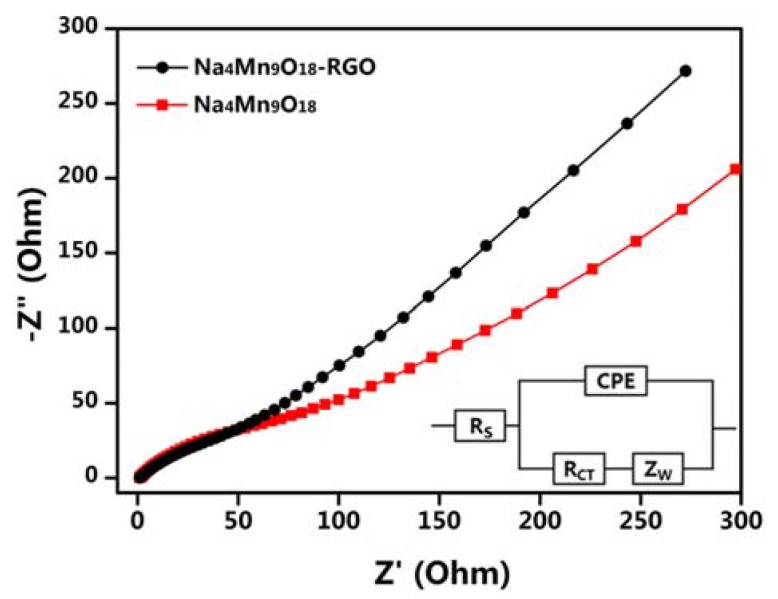
EIS of the Na_4_Mn_9_O_18_-RGO and Na_4_Mn_9_O_18_ electrodes and the equivalent circuit model of plot fitting (inset).

Nyquist plots of the Na_4_Mn_9_O_18_-RGO and Na_4_Mn_9_O_18_ electrodes were produced with a frequency range of 10^5^–0.01 Hz and are presented in Figure 8. The inset of Figure 8 is a simple equivalent circuit model applied to fit the EIS. 

The *R*_S_ is the electrolyte resistance of cell components, *R*_CT_ is associated with the charge transfer procedure at the electrode-electrolyte interface, *Z*_W_ is the Warburg impedance that is associated with sodium-ion diffusion in the electrode, and CPE is associated with the double layer capacitance. It can be seen that the charge transfer resistance (*R*_CT_) of the Na_4_Mn_9_O_18_-RGO electrode is a little smaller than that of Na_4_Mn_9_O_18_. Additionally, the *Z*_W_ of the Na_4_Mn_9_O_18_-RGO electrode has a bigger slope, which indicates enhanced sodium-ion diffusion. Our EIS measurements demonstrated that the RGO sheets are beneficial to improving electrochemical performance.

## 4. Conclusions

In summary, the Na_4_Mn_9_O_18_-RGO particles have been successfully synthesized by a hydrothermal soft chemical reaction and via spray drying. An aqueous sodium ion battery using metallic Zn and Na_4_Mn_9_O_18_-RGO as the negative and positive electrodes, respectively, has been developed. Compared to the Na_4_Mn_9_O_18_ electrode, the Na_4_Mn_9_O_18_-RGO electrode offered a larger discharge capacity of 58.9 mAh·g^−1^ at 4 C, even after 150 full cycles due to the network structure of RGO. The superior electrochemical performance demonstrates that Na_4_Mn_9_O_18_-RGO can be a promising material for a safe and efficient ASIB.
